# Large-effect loci in a duplicated genomic region modulate reproductive phenology in salmon

**DOI:** 10.1101/2024.03.30.587279

**Published:** 2026-07-31

**Authors:** Patrick D. Barry, Natasha Howe, Gregory L. Owens, Diana S. Baetscher, Katie D’Amelio, Scott Vulstek, Joshua Russell, Elizabeth Lee, Andrew Barclay, Anthony J. Gharrett, David A. Tallmon, Wesley A. Larson

## Abstract

Whole genome duplication (WGD) provides evolutionary opportunities to increase biological complexity and phenotypic diversity by selective retention of duplicated genes. We identify a small genomic region in salmon that governs complex life history variation and has diversified across four WGDs to fine-tune trait expression. Within this region, three large-effect loci on three chromosomes modulate migration timing across four species - a critical trait optimizing fitness by synchronizing reproduction and environmental conditions. Life history and genome resequencing data support intraspecific evolution of polymorphisms related to early- and late-reproducing phenotypes. We demonstrate that the repeated evolution of loci underlying reproductive timing carries the signature of WGD-facilitated adaptive evolution. This adaptation is driven by the subsequent subfunctionalization of genes within the highly conserved HPG axis, which regulates vertebrate reproduction.

## Full Text Availability

The license terms selected by the author(s) for this preprint version do not permit archiving in PMC. The full text is available from the preprint server.

